# Long QT-Syndrome With Torsades de Pointes Managed Considering Financial Constraints Faced by the Patient

**DOI:** 10.7759/cureus.15892

**Published:** 2021-06-24

**Authors:** Ramesh Patel, Sandeep Aggarwal, Pal Satyajit Singh Athwal, Sandeep Randhawa, Sukhmanii Kahlon

**Affiliations:** 1 Cardiology, Geetanjali Medical College and Hospital, Udaipur, IND; 2 Cardiovascular Division, University of Minnesota School of Medicine, Minneapolis, USA; 3 Department of Infectious Diseases, University of Washington Valley Medical Center, Renton, USA; 4 Internal Medicine, University of Hawaii, Hawaii, USA; 5 Internal Medicine, Medical University of the Americas, Camps, KNA

**Keywords:** long qt syndrome, life threatening arrhythmia, congenital long-qt syndrome, sudden cardiac, torsades de pointes

## Abstract

Long QT syndrome (LQTS) is a rare arrhythmogenic condition characterized by abnormally long QT intervals on an electrocardiogram. The prevalence varies between 1 in 3000 and 1 in 10,000 but often remains undiagnosed. It is responsible for 3000 to 4000 sudden deaths among children and adults in the United States alone. LQTS can lead to torsades de pointes which is seen as twisting of QRS complex on electrocardiogram. We report a case of a 35-year-old patient with LQTS who presented with syncope and was found to have torsades de pointes. After acute management the patient was advised for automatic implantable cardioverter defibrillator (AICD) but because of financial constraints, she was placed on beta-blockers and permanent pacemaker.

## Introduction

The long QT syndrome (LQTS) is characterized by abnormally prolonged QT interval and T-wave abnormalities leading to life-threatening arrhythmias such as torsades de pointes and is one of the leading causes of sudden death. The prevalence ranges between 1 in 3000 and 1 in 10,000 but most of the cases remain undiagnosed [1]. Congenital LQTS is caused by mutations in genes such as KCNQ, KCNH2, SCNA5, KCNE1. Based on these mutations LQTS is divided into many subtypes. On the other side, acquired cause of long QT on electrocardiogram can be due to electrolyte imbalance or drugs. Inherited LQTS involves Jervell and Lange-Nielsen syndrome which is an autosomal recessive disorder with long QT intervals and sensorineural deafness. Others included are Romano-Ward syndrome, Andersen-Tawil syndrome, and Timothy syndrome [2,3]. The patient usually presents with presyncope or syncope but sudden cardiac death can be the first presentation for this syndrome. Emergency measures include electric cardioversion, beta receptor blockers, potassium supplementations, stellate ganglion blockade, and implantable cardioverter-defibrillator.

## Case presentation

**Figure 1:**
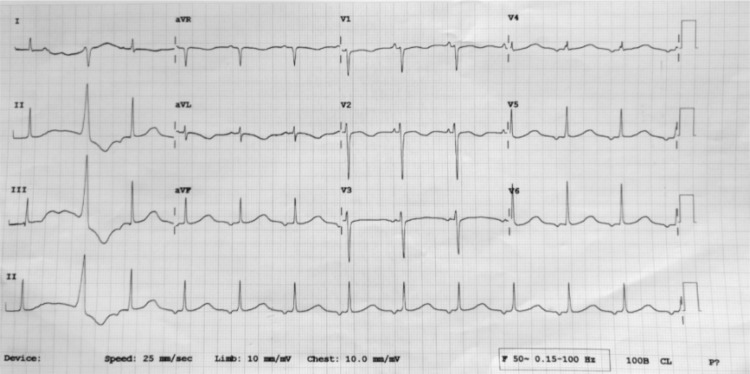
Electrocardiogram showing long QT intervals.

A 35-year-old female was admitted with a history of recurrent loss of consciousness for which she had consulted a neurologist and then referred to a cardiologist. On admission, she was having intermittent ventricular tachycardia with significant hemodynamic compromise, requiring recurrent DC cardioversion. She was started on amiodarone and injection heparin immediately. She had no family history of QT prolongation, arrhythmias, QT-prolonging drugs, or sudden cardiac death. Evaluation of ECG showed significant QT prolongation (Figure 1).

**Figure 2:**
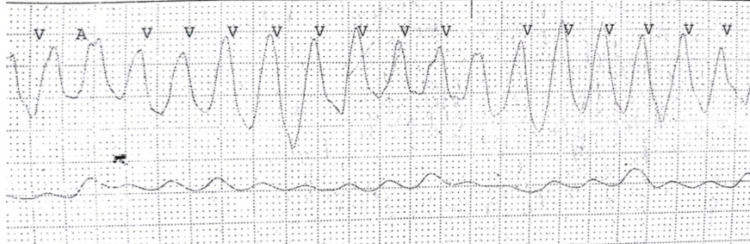
Torsades de pointes

The corrected QT interval was 580 msec and VT was typical polymorphic ventricular tachycardia with changing axis and amplitude indicating it was torsades de pointes (Figure 2). Labs revealed no acquired causes (Table 1) of long QT syndrome and the patient reported no history of QT prolonging drugs or any medical history of diseases causing QT prolongation.

**Table 1:** Causes of acquired QT prolongation and torsades de pointes

1) Electrolyte disbalance like low potassium, calcium, or magnesium
2) Drugs such as antiarrhythmics, antibiotics, antipsychotics, antihistamines, citrate (post blood transfusion), methadone, cocaine
3) Cardiac conditions- Myocardial infarction, myocarditis, bradycardia
4) Endocrinopathies such as hypothyroidism, hyperparathyroidism, pheochromocytoma
5) Intracranial disorders
6) Nutritional disorders- Anorexia nervosa, starvation, Celiac disease

Diagnostic criteria in the form of Schwartz score are used for LQTS as shown in Table 2. Calculated Schwartz score in our patient was >4, indicating high probability of congenital LQTS.

**Table 2:** Diagnostic criteria for long QT syndrome (LQTS) Definite LQTS is defined by an LQTS score ≥4. Scoring: ≤1 point, low probability of LQTS; 2–3 points, intermediate probability of LQTS; ≥4 points, high probability of LQTS.

ECG findings		Score
A) QTc	≥480 ms	3
	460–479 ms	2
	450–459 ms (in males)	1
B) Torsade de pointes		2
c) T wave alternans		1
D) Notched T wave in three leads		1
E) Low heart rate for age		0.5
Clinical History		
A) Syncope		
with stress		2
without stress		1
B) Congenital Deafness		0,5
Family history		
A) Family history with LQTS		
B) Unexplained sudden cardiac death below age 30 among immediate family members		0.5

2D echocardiography showed normal LV function. Intravenous amiodarone was immediately stopped and intravenous lidocaine was continued (both class IA and III are associated with QT prolongation). Despite this the patient was having hemodynamically significant torsades de pointes requiring repeated shocks. To curtail sympathetic surge we sedated her and intubated and put her on mechanical ventilation.

More than 25 shocks were given by this time, but there was no improvement in the episodes of torsades de pointes. We reviewed the literature and planned to add a beta-receptor blocker in presence of pacing. So we put a temporary pacemaker through the right femoral vein in the right ventricular apex and kept pacing at 100 per minute and added metoprolol tartrate 25 mg 12 hourly. After this, there was no episode of torsades de pointes and the patient improved significantly.

She was extubated and all of her infusions were stopped. She was advised for automatic implantable cardioverter defibrillator (AICD) as further management, but because of economic constraints, the patient refused. But looking at the viability, she had achieved on temporary cardiac pacing and beta-blocker, so we put her on a permanent pacemaker and metoprolol tartrate 25 mg twice daily. She is stable with no episode of VT after one month of follow-up.

## Discussion

LQTS is a rare inherited arrhythmogenic disorder which can lead to life-threatening torsades de pointes and is also one of the leading causes for sudden cardiac death. Prolonged QT interval on electrocardiogram is due to ion channel dysfunction which is responsible for the regulation of action potential. Inherited LQTS involves well-known syndromes like Jervell, Lange-Nielsen, and Romano-Ward but none of these fits in with our patient making it an isolated case of LQTS [4]. KCNQ1, SCN5A, and KCNH2 are the most common genes associated with LQTS [5]. Most of the patients present with syncope or presyncope as in our case.

Schwartz scoring system is used for the clinical diagnosis of LQTS which is based on ECG changes such as QTc interval, T wave changes along with clinical features and family history. Schwartz score of >3 can be used to determine which cases need to be genetically tested for mutations [6]. Diagnosis is based on the first exclusion of acquired causes of QT prolongation on electrocardiogram. Even with high Schwartz score we were unable to get the genetic test done in our patient due to financial constraints.

We managed this case with a long-term beta-blocker and permanent pacemaker even though an ICD was indicated due to the high M-FACT score which was 3 in our patient.

M-FACT risk score (Table 3) is based on Qtc interval, events on or off therapy, age at implant, prior aborted cardiac event [6]. Implantable cardioverter-defibrillator (ICD) remains a Class 1 in patients with a diagnosis of LQTS who are survivors of a cardiac arrest [7]. The patient refused the ICD because of financial constraints - cost of the ICD is around $5000 in India. Single chamber pacemakers are way cheaper and costs around $1900.

**Table 3:** M-FACT risk score

	-1	0	1	2
Event free on therapy > 10 years	yes			
QTc		<500	>500-<550	>550
Prior aborted cardiac arrest (ACA)		No	Yes	
Event on therapy		No	Yes	
Age at implant		>20 years	<20 years

Management modalities involve beta-blockers, left cardiac sympathetic denervation, implantable cardioverter defibrillator, and gene-specific therapy. Beta-blockers are used to curb the adrenergic tone but are not effective in all the LQTS subtypes. Propranolol or nadolol was found to be better as compared to metoprolol for long-term management of LQTS in a study by Chockalingam et al. [8]. Failure of beta-blockers can be due to omission of the drug or use of other drugs which prolongs QT by an uninformed physician [9]. A study by Schwartz et al. demonstrated a significant reduction of cardiac arrest and syncope after left cardiac sympathetic denervation but was less effective on long-term follow-up [10]. Implantable cardioverter-defibrillator (ICD) if not implanted can lead to tragic outcomes, indicated in patients with high QT interval and an episode of the hemodynamic episode as in our patient but because of financial constraints the patient was managed with any ICD.

Torsade de pointes is due to QT prolongation which can lead to life-threatening ventricular fibrillation and sudden cardiac death. First line pharmacological agent is magnesium which stabilizes the cardiac membrane. Hemodynamic compromised patients should be electrically cardioverted [11].

## Conclusions

Long QT syndrome (LQTS) is a rare syndrome characterized by long QT intervals and is one of the causes of sudden cardiac death. We report a case of LQTS in 35-year-old female who was found to have torsades de pointes with significant hemodynamic compromise and a corrected QT interval of 580 msec. Implantable cardioverter-defibrillator (ICD) remains a Class 1 in patients with a diagnosis of LQTS who are survivors of a cardiac arrest. If ICD is not implanted, it can lead to potential sudden cardiac death. We implanted a single chamber pacemaker along with beta-blockers as an alternative. This case represents the tip of the iceberg of problems faced by patients due to financial constraints, especially in developing countries. The physician should be very vigilant while using drugs that prolong QT interval to prevent any catastrophic event. This case also represents the use of pacing with beta-blocker in a case of Long QT syndrome.
